# In this month’s Bulletin

**DOI:** 10.2471/BLT.26.000426

**Published:** 2026-04-01

In the editorial section, Meg Doherty et al. (210) detail the history and contributions of WHO’s collaborating centres.

## Argentina, Australia, China, Costa Rica, Denmark, Germany, India, Japan, Mexico, Netherlands, Norway, Republic of Korea, Russian Federation, Saudi Arabia, South Africa, Sweden, Switzerland, Thailand

### Surveillance of antimicrobial resistance

Anne Harant et al. (259–273) detail the functions of a global network of WHO collaborating centres.

## Bangladesh 

### Hepatitis C infections among Rohingya refugees

Giancarlo Ceccarelli et al. (282–284) describe a public health response programme.

## Cambodia

### Provision of prosthetic limbs

Thearith Heang et al. (213–222) evaluate outcomes.

## Cameroon

### Cholera control targets

Armelle Ngomba et al. (223–233) map priority areas.

## Global

### Eligible blood donors

Luce Mosselmans et al. (234–245) examine the data on exclusion criteria.

### Benefit–cost analyses of environmental and climate-health interventions

Frank Pega et al. (246–258) calculate value of lives saved. 

### Hip and knee replacements worldwide 

Peter G Delaney and Nicolas S Piuzzi (274–281) make the case for arthroplasty as essential surgery.

